# The Profiling and Role of miRNAs in Diabetes Mellitus

**DOI:** 10.33696/diabetes.1.003

**Published:** 2019

**Authors:** Michael Kim, Xiaokan Zhang

**Affiliations:** Department of Medicine, Division of Cardiology, Columbia University Medical Center, New York, NY, USA

**Keywords:** Diabetes Mellitus, microRNA

## Abstract

Diabetes mellitus (DM), a complex metabolic disease, has become a global threat to human health worldwide. Over the past decades, an enormous amount of effort has been devoted to understand how microRNAs (miRNAs), a class of small non-coding RNA regulators of gene expression at the post-transcriptional level, are implicated in DM pathology. Growing evidence suggests that the expression signature of a specific set of miRNAs has been altered in the progression of DM. In the present review, we summarize the recent investigations on the miRNA profiles as novel DM biomarkers in clinical studies and in animal models, and highlight recent discoveries on the complex regulatory effect and functional role of miRNAs in DM.

## Introduction

Diabetes mellitus (DM) is an age-related metabolic disorder affecting 347 million people in modern society. Expanding its prevalence beyond developed countries, DM has emerged as a global public health issue associated with a high morbidity and mortality. According to an estimation by the International Diabetes Federation (IDF), the global population affected by diabetes will reach 552 million by the year 2030 [1]. DM is a complex disease characterized by an insufficient secretion of insulin from pancreatic β-cells that prevents the normal maintenance of blood glucose homeostasis. There are two major forms of diabetes. Type 1 diabetes mellitus (T1DM) is due to lack of insulin hormone production from pancreatic β-cells, while type 2 diabetes mellitus (T2DM) results from ineffective insulin response [2]. The clinical manifestations of DM restrict its timely diagnosis, which may be delayed by several years. This delayed prediction often results in chronic complications, including cardiovascular disease associated with DM [3].

According to a recent estimation, there are more than 2000 mature human miRNAs that have been recognized since the first miRNA was identified in 1993 [4]. MiRNAs have emerged as major regulators of gene expression and are involved in the onset and progression of various diseases [5–7]. A growing body of evidence indicates that a specific set of miRNAs has an altered expression profile in the progression of DM [8–11], making these biomolecules potential biomarkers for the prognosis, diagnosis and management of disease. Individual miRNAs or whole miRNA clusters associated with diabetes have been observed to be dysregulated in expression and activity, therefore generating a rising interest in their therapeutic use as clinical targets. The diverse role of miRNAs in the etiology and pathogenesis of DM has been widely explored [12–14], and new findings are continuously emerging. In this present review, we summarize the recent findings on the potential role of miRNAs as biomarkers in the settings of diabetes, and the dysregulation of miRNAs and their molecular targets, to obtain a better understanding of the application of miRNAs in the development of DM.

## Role of miRNAs in Regulating Gene Expression

MiRNAs are small single-stranded non-coding RNAs involved in nearly every stage of biological processes. They are best known to regulate gene expression at the post-transcriptional level [15]. Indeed more than 60% of protein-coding genes in mammalian cells are conserved targets of miRNAs, which partially base pair with the 3’UTR of the mRNA targets through their 5’-proximal seeding region [16]. This complex targeting process is tightly regulated under different cellular conditions, and eventually leads to the reduced expression of the target gene [17,18].

**Figure 1:**
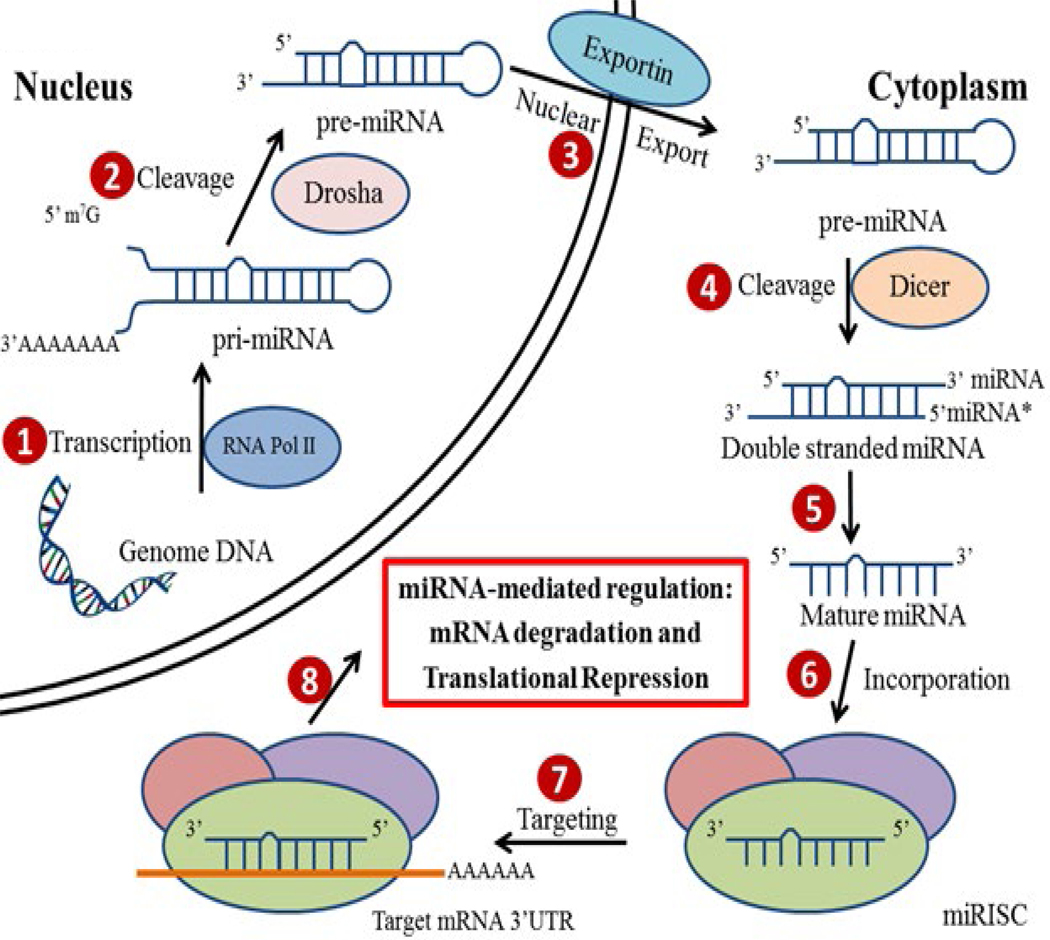
Biogenesis and function of miRNAs in the regulation of gene expression. The pri-miRNAs are transcribed in the nucleus by RNA Pol II (1). Then pre-miRNAs are generated by the Rnase III enzyme Drosha mediated cleavage (2). The pre-miRNAs are transported into the cytoplasm through Exportin (3), cleaved by Dicer to yield double stranded miRNAs (4), and are further processed into single stranded mature miRNAs (5). Mature miRNAs are incorporated into miRISC (6), which is the functional units for targeting mRNAs (7) and reducing gene expression through either mRNA degradation or translational repression (8).

MiRNAs are encoded within the genomes of various species, ranging from protozoans to mammals [19]. Even though most of the mature miRNAs are approximately 22 nucleotides in length, they are first transcribed into long precursor molecules (~1000 nucleotides in length) called primary miRNAs (pri-miRNAs) by RNA polymerase II enzyme. Then with the help of nuclear microprocessor complex consisting of RNase III enzyme Drosha, the pri-miRNAs are further transformed into smaller (<100 nucleotides in length), hairpin-shaped precursor miRNAs (pre-miRNAs) and transported from nucleus into the cytoplasm [20]. In the cytoplasm, the pre-miRNAs are cleaved by RNase III-type endonuclease Dicer, and then are further processed into single stranded mature miRNAs [21]. However, the miRNAs alone are not functional until they are incorporated into the miRNA-induced silencing complex (miRISC). With the recruitment of a group of RNA binding proteins (RBPs), miRISCs are delivered to their mRNA targets in order to regulate the expression of these genes [22] (Figure 1).

Mechanistically, miRNAs regulate gene expression via two distinct and independent pathways, either through mRNA deadenylation activation, which subsequently leads to transcript degradation, and/or through translational repression [22–24]. In previous years, many studies have shown that the elements of the miRISC can functionally interact with a group of RNA binding proteins, facilitating the accurate anchor of the miRISC to the cis-acting regulatory sites of mRNA targets [25]. Interestingly, each mRNA could have multiple miRNA targeting sites at the 3’UTR, whereas each miRNA could base-pair with multiple mRNA targets, resulting in a complex miRNA-mediated gene expression regulatory network [23]. Thus, miRNAs demonstrate a strong role as contributors to the development of DM in humans.

## MicroRNA Profiling in Diabetes

In the past decades, the specific profiling of miRNAs has been studied extensively in both blood and pancreatic islets, demonstrating a signature of miRNA alteration is implicated in the context of DM.

## Circulating miRNAs as biomarkers in predicting diabetes

In recent years, multiple studies have suggested that circulating miRNAs are correlated with various human diseases, including diabetes. Profiling miRNA content in circulation may reflect the dynamic changes of circulating cells in response to disease states. Therefore, their potential as biomarkers for the prediction and diagnosis of DM has become increasingly appreciated.

A large number of studies reporting the dysregulation of miRNAs in the serum/plasma of patients with diabetes have emerged in the past decade. A recent systematic review has identified eleven circulating miRNAs consistently dysregulated in T1D patients compared to controls: miR-21–5p, miR-24–3p, miR-100–5p, miR-146a-5p, miR-148a-3p, miR-150–5p, miR-181a-5p, miR-210–5p, miR-342–3p, miR-375 and miR-1275 [26]. One interesting study compared the expression levels of serum miRNAs from new onset T1D children and age-matched healthy controls identified twelve up-regulated miRNAs in T1D patients, including miR-24, miR-25, miR-26a, miR-27a, miR-27b, miR-29a, miR-30a-5p, miR-148a, miR-152, miR-181a, miR-200a and miR-210 [27]. Another study providing additional information on the profile of circulating miRNAs in children with recent onset of T1D has detected a significant increase in miR-144–5p, miR222–3p, miR-345–5p and miR-454–3p levels in the serum of children with T1D versus non-diabetic controls [28]. Additionally, the plasma levels of miR-21, miR-24, miR29a, miR-30d, miR-34a, miR-126, miR-146, and miR148a showed a significant up-regulation in one study comparing 16 adults T1D patients with 27 healthy controls [29]. Evaluated by qRT-PCR, higher levels of miR-21 and miR-210 were confirmed by Osipova et al., in the plasma of T1D patients [30]. Nabih et al., has also revealed that miR-181a expression was up-regulated in the serum of subjects with T1D [31]. As it is not uncommon in a rapidly evolving field, there have been numerous conflicting reports regarding the expression signature of miRNAs in T1D patients. Increased miR-375 in the serum of subjects with T1D has been shown in several studies [29,32], while decreased miR-375 was also reported in the serum of T1D children compared to age-matched controls [33]. Among these miRNAs reviewed above, miR-21, miR-24, miR-148a, miR-181a-5p and miR-210–5p have been confirmed to be up-regulated in T1D in more than one independent study. This consistent data strongly supports the potential of miRNAs as circulating biomarkers of T1D (Table 1).

In a global profile study focusing on T2D circulating miRNAs, approximately 70 miRNAs showed elevated levels and about 100 miRNAs showed reduced levels in blood samples of T2D patients [34]. A meta-analysis confirmed 40 significantly dysregulated miRNAs in T2D patients, and highlighted that circulating miR-29a, miR34a, miR-103, miR-107, miR-132, miR-142–3p,miR-144 and miR-375, levels may serve as potential biomarkers for T2D [35]. In addition, the most down-regulated miRNA is miR-126a, which has been validated by several other studies in both the serum [36] and plasma [37–41] of T2D patients. Interestingly, when plasma miR-126 levels were determined in three study groups, including a healthy normal control, T2D-susceptible, and T2D patients, miRNA-126 was significantly reduced in both susceptible and T2D individuals, indicating miR-126 is tightly associated with the manifestation of T2D and could be a potential circulating biomarker for the early identification of individuals susceptible to T2D [40]. Another T2D-related miRNA signature change evaluated by several groups is miR-146a. Surprisingly, the expression pattern of miR-146a determined in different experiment settings is not consistent. Some showed miR146a levels to be elevated in serum [42] and plasma [43], while other studies demonstrated lower levels of miR-146a in serum [44,45] or whole blood [34] from T2D patients. While differences in study populations might have caused this discrepancy, future research will require additional effort to understand the subtleties of the regulation of this miRNA. In serum, age-based comparisons between a control group with normal glucose tolerance and T2D individuals demonstrated significantly increased levels of miR-9, miR-15b, miR-27a, miR-29a, miR-30d, miR34a, miR-124a, miR-146b, miR-150, miR-192, miR-320a, miR-375, miR-486, miR-571, miR-661, miR-770, miR892b and miR-1303 [42,46–48], and decreased expression levels of let-7i, miR-23a, miR-96, miR-186, miR-191, miR192, miR-486 [44]. Altered expression levels of miRNAs in plasma have also been assessed, in which miR-28–3p was highly expressed [38], while miR-15a, miR-21–5p, miR29b, miR-191, miR-200b and miR223 displayed reduced levels [38,41,49] in T2D patients. Additional studies revealed that miR-29a, miR-144, miR-150, miR-192, and miR-320 were up-regulated, whereas miR-15a, miR30d and miR-182 were down-regulated in whole blood evaluations [34,50], and the expression of miR-103b is decreased in platelets of patients with T2D [51] (Table 1).

Although human studies provide clinically disease relevant information to understand the onset of diabetes, major limitations must be considered, such as the limited amount of material from donors, relatively small cohort numbers, and difficulties in finding of non-diabetic controls matched for age, gender and ethnicity. Moreover, the interpretation of these data can be affected by the delayed diagnosis of the disease and the use of various medications on these patients. As a result, the use of animal models can provide valuable insight to understanding the manifestation of diabetes and its underlying molecular mechanisms. Streptozotocin (STZ) is a chemical that destroys insulin-producing cells and is commonly used for the generation of T1D phenotype in mice, while nonobese diabetic (NOD) mice is another rodent model for T1D due to insulitis, a leukocytic infiltrate of the pancreatic islets. The level of miR-375 in plasma showed a significant increase in both STZ-treated mice and NOD mice before diabetes onset, indicating miR-375 could be a suitable blood marker to predict diabetes [52]. In one study on high fat diet fed (HFD) T2D rat model, miR-30d, 146a and miR182 showed reduced expression, while miR-29a, miR-144, miR-150, miR-192 and miR-320a were found to be highly elevated [34]. The Zucker diabetic fatty (ZDF) rat model reflects many characteristics of human conditions, making it an ideal model to observe the natural progression of T2D [53]. In the ZDF rat model, several miRNAs were found to be elevated over the course of T2D, such as miR-122, miR133, miR-210 and miR-375, while others miRNAs including miR-140, miR-151–3p, miR-185, miR-203, miR-434–3p and miR-450a were found to be decreased [54]. The db/db mouse, another rodent T2D model with leptin receptor deficiency, obesity, insulin resistance, hyperglycemia and hyperinsulinemia, is also used to investigate circulating miRNA profiling changes [55]. The serum miR-16b, miR146b and miR-486 showed significantly higher levels in db/db mice compared to those in age-matched male C57BL/6J mice [46] (Table 2).

## miRNAs Signature in Pancreatic Islets

Many miRNAs are known to be cell-type or tissue specific. It has been shown that a group of islet-enriched miRNAs participate in the development of the pancreatic islet, the regulation of islet mass, insulin secretion and β-cell proliferation and apoptosis, thus suggesting an important role of these miRNAs in pancreatic islet function. For example, miR-7, miR-9, miR-375 and miR-376 have been shown to be expressed at high levels in the human pancreas during the development and maturation of pancreatic islets [56–58]. To identify the individual miRNAs and how their expression patterns are dynamically regulated throughout the development of diabetes may be of diagnostic and therapeutic interest. Although changes in the profile of miRNAs in pancreatic islets are less investigated than those in circulating miRNAs, scientific knowledge in this area is rapidly increasing.

MiRNA expression profiles between human islets isolated from donors with diabetes and non-diabetic subjects provide valuable insights into the discovery of miRNAs associated with diabetes. In one recent study, miR-125a-5p showed elevated expression levels in donors with T1D compared to donors without diabetes [59]. The miRNA expression profile in T2D has also been widely analyzed in human pancreatic islets. A meta-analysis performed by Zhu et al., suggested the dysregulation of two highly pancreas-specific miRNAs, miR-199a-3p and miR-223, could potentially be tissue biomarkers of T2D [35]. MiR-124a expression was found to be significantly increased [60], and miR-187 hyperexpression was identified in human islets tissue from individuals with T2D versus matched controls [61]. On the other hand, some miRNAs have been found to have lower expression levels. MiR-7a, which regulates pancreatic β-cell function, showed a reduced expression in T2D islets [62]. Tattikota et al., additionally showed a dramatic down-regulation of miR-184 in 12 diabetic individuals [63] compared to pancreatic islets from 15 non-diabetic donors. A cluster of miRNAs highly and specifically expressed in human β-cells, including miR-127, miR-136, miR-369, miR-411, miR-432, miR-487, miR-495, miR-543, miR-589, miR-655 and miR-656, is significantly decreased in islets from T2D organ donors [64] (Table 3).

The miRNA expression signature in pancreatic islets has been explored in animal models as well. In one recent study, 64 up-regulated and 72 down-regulated pancreatic miRNAs were detected in streptozotocin (STZ)-induced T1D mice compared to normal controls via a miRNA microarray, and several of them, including let-7a-5p, let-7b5p, let-7f-5p, miR-7a-5p, miR-7b-5p, miR-26a-5p, miR26b-5p, miR-27a-3p and miR-148b-3p, were confirmed by qRT-PCR to have decreased expression levels in diabetic mice [65]. By using another T1D murine model, Ma et al, has shown the down-regulated expression of miR-26a-5p in pancreatic tissues from NOD mice [66]. Other studies have also been done to evaluate miRNA expression pattern in pancreas from rodent models of T2D. A global miRNA profile analyzed in the islets of non-obese T2D model Goto-Kakiz (GK) rats, which spontaneously develop T2D unrelated to obesity [67], identified 30 miRNAs with different expression patterns compared to Wistar controls; GK rat miRNAs clustered into 6 miRNAs with lower expression and 24 miRNAs with higher expression [68]. Another study compared the miRNA expression signature in a T2D rat model and showed that in pancreatic tissue, miR-29a, miR-144, miR-150, miR-192 and miR-320a were highly up-regulated, while miR-30d, miR-146a and miR-182 were highly down-regulated [34]. Regarding T2D mice model, elevated levels of the miR-200 family, which consists of miR-141, miR-200a, miR-200b, miR-200c and miR-429, were observed in islets of db/db diabetic mice at 12 weeks of age [69]. Additional studies showed the expression levels of let-7b, miR-21, miR-34a, miR-132, miR-146, miR-199a-3p and miR-199a-5p to be strongly induced in the pancreatic islets of diabetic mice [70–72]. The miRNAs reported to have reduced expression levels, detected by qRT-PCR in diabetic islets, are miR-30d, miR-184, miR-203, miR-210, miR-338–3p and miR-383 [71–73]. Interestingly, reduced miR-184 was also observed in the islets of 12-week-old leptin receptor-deficient db/db mice, which showed a similar loss of expression in human diabetic individuals [63]. Diet-induced obesity (DIO) mice, which are fed a HFD to induce obesity and insulin resistance, are a valuable model of pre-diabetes or the early phases of T2D [74]. A loss of expression of mature miR-184 in the islets of mice on a HFD was observed [63], making miR-184 a consistently down-regulated miRNA in more than one mouse model of obesity and insulin resistance. qRT-PCR analysis also revealed elevated levels of miR-132, miR-199a-3p and miR-199a-5p, and decreased levels of miR-7a, miR-184, miR-203, miR-210 and miR383 in islets of DIO mice [62,72] (Table 4).

Although the signature pattern of miRNAs in diabetes has been widely studied, the biological correlation between circulatory miRNA and pancreatic islets miRNA expression has not been established. To reveal the detailed molecular mechanisms underlying the regulation on the changes of miRNAs in the global large scale studies is a primary step towards using them as predictive and diagnostic biomarkers in real clinical practice.

## Role of miRNAs in Diabetes

Various studies have shed light on the miRNA-mediated pathways controlling glucose homeostasis. The regulation of glucose by islet-enriched miRNAs principally occurs through the production and secretion of insulin and the survival and proliferation of β-cells. The aberrant expression and activity of these pancreatic miRNAs may have significant consequences on these regulatory pathways, potentially driving clinical hyperglycemia associated with T1D and T2D (Table 5).

### Secretion of insulin

Islet-enriched miRNAs act on a diverse array of downstream targets influencing the secretion of insulin. One review has established a profile of human islet derived miRNAs that control insulin secretion by targeting the exocytosis machinery of the β-cell. Notably, all of the miRNAs profiled in this study inhibit the secretion of insulin, suggesting an evolutionarily conserved role of islet miRNAs in preventing lethal hypoglycemia [75].

Through qRT-PCR methods, miR-375 was found to be the most highly expressed miRNA in human pancreatic islet and is known to have a well-defined role in down-regulating insulin secretion [75]. MiR-375 is also highly expressed in murine insulinoma MIN6 cells and one study revealed that miR-375 targets the 3’ UTR of the Myotrophin (Mtpn) mRNA [58]. Mtpn is actively involved in the cytoskeletal remodeling process by depolymerizing actin filaments and allowing for the fusion of insulin vesicles at the β-cell membrane [76]. A miR-375-mediated regulation of the Mtpn exocytosis pathway helps to explain an observed decrease in glucose-stimulated insulin secretion (GSIS) in this model [58]. Mtpn is also known to up-regulate the nuclear transcription factor NF-κB, which may subsequently activate the expression of proteins involved in the trafficking of insulin vesicles to the membrane [77]. Supporting this study’s findings, another group developed an in vitro miR-375 overexpression system in mouse insulinoma Nit-1 cells and verified a reduction in GSIS via the miR-375/Mtpn targeted interaction [78].

MiR-124a and miR-96 have also been found to influence the exocytosis machinery in MIN6 cells [79]. Interestingly, miR-124a increases insulin secretion at basal glucose levels while decreasing GSIS. The variable expression of pro-secretory proteins in a miR-124a overexpression system can help explain these findings: SNAP25, Rab3A, and synapsin-1A levels increased while Rab27a and Noc2 levels decreased. Because Rab27a is a GTPase that facilitates the transport of vesicles to the cell membrane and was specifically found to be a direct target of miR124a, the diminished expression of Rab27a helps to explain the reduced cellular capacity to respond to high glucose conditions. MiR-96 also decreases GSIS by indirectly inhibiting Noc2 expression while increasing the expression of granuphilin, a protein that inhibits insulin exocytosis (79).

The ATP-binding cassette transporter A1 (ABCA1), a cholesterol efflux facilitator, is also implicated in decreased insulin secretion in murine models. MiR-33a and miR-145 were found to target ABCA1, mediating the accumulation of cholesterol in murine islets and decreasing insulin secretion [80,81].

MiR-7 has been shown to down-regulate GSIS by modulating the distal stages of the insulin exocytosis pathway. Mechanistically, miR-7 represses the expression of SNCA and concomitantly inhibits the formation of the SNARE ternary complex, blocking the exocytosis of insulin granules already docked at the β-cell membrane [62].

### Production of insulin

In addition to targeting components of the secretory machinery in the β-cell, miRNAs can also influence the production of insulin. The role of miR-375 in glucose homeostasis extends beyond insulin trafficking as it also targets 3’-phosphoinositide-dependent protein kinase-1 (PDK1), a key component of the phosphatidylinositol 3-kinase (PI3K) cascade [82]. Reduced PDK1 levels are associated with decreased insulin gene expression in response to glucose stimulation. Interestingly, high glucose conditions yielded a decrease in precursor miR375 expression and an associated increase in PDK1 and insulin levels [82].

MiR-204 has also been found to play a negative role in insulin production. Thioredoxin-interacting protein (TXNIP), a regulator of redox states in the β-cell, induces the expression of miR-204. Mechanistically, TXNIP decreases the phosphorylation and activity of signal transducer and activator of transcription 3 (STAT3), a known repressor of the miR-204 promoter. TXNIP is upregulated in diabetes and a concomitant increase in miR204 expression allows for the increased direct targeting and degradation of MAFA, an established transcription factor for insulin [83]. Additionally, miR-204 directly targets the 3’ UTR of Glucagon-like peptide 1 receptor (GLP1R) and down-regulates GSIS, demonstrating another connection between TXNIP and glucose homeostasis that is mediated by miR-204 [84].

### Beta cell proliferation and survival

Many pancreatic miRNAs also regulate β-cellular pathways driving proliferation and survival, as well as β-cell destruction induced by the presence of proinflammatory cytokines. Through the aforementioned PI3K pathway, miR-375 not only down-regulates PDK expression but also plays an inhibitory role in β-cell proliferation and survival. Supporting this notion, INS-1E cells transfected with a precursor form of miR-375 showed a 25% reduction in proliferation and a 20% reduction in viability, compared to INS-1E cells transfected with a control vector; [methyl3H] thymidine incorporation during DNA synthesis also decreased by 20% in INS-1E cells transfected with precursor miR-375, verifying the aforementioned decrease in cellular proliferation [82]. However, the genetic deletion of miR-375 expression in murine models similarly impaired proliferation and diminished β-cell mass, suggesting that an intermediate and steady state miR-375 level is optimal to promote the adequate survival and proliferative capacity of β-cells [76].

MiR-21 is also involved in the cellular machinery regulating β-cell number [85,86]. Because miR-21 is known to play a pro-proliferative role in β-cell survival, one group overexpressed miR-21 in INS-1 cells and interestingly observed a net decrease in β-cell number despite confirming an increase in cell proliferation [85]. Attributing this net decrease to a hyperactive flux through the cell cycle and a concomitant activation of checkpoint-mediated apoptosis, they indeed found an irregular expression of two cell cycle genes involved in the first apoptosis checkpoint of the G1 phase. The proinflammatory cytokine pathway involving NFκB up-regulates the expression of miR-21, which may play a role in increasing NO synthesis and β-cell apoptosis [85]. In contrast, another study overexpressed miR-21 in MIN6 cells and did not ascertain any effect on cell survival, but did find that decreasing miR-21 expression promoted apoptosis [86]. MiR-34a is known to play the opposite role of miR-21 by targeting protein silent information regulator 1 (SIRT1) and increasing p53-mediated apoptosis. Indeed, the net effect of miR-34a knockdown was an increase in β-cell mass [85].

MiR-200 is strongly linked to β-cell pathology in both T1D and T2D. Specifically, miR-200 suppresses the antiapoptotic and stress-resistance pathways that include Dnajc3, a β-cell heat shock protein, and Xiap, a caspase inhibitor. MiR-200 also facilitates the activation of the tumor suppressor protein Trp53, fostering the expression of pro-apoptotic genes [87].

## Summary

Pancreatic miRNAs act through a diverse series of pathways regulating the biological development and function of the β-cell. Disrupting the miRNA expression profile in β-cells has shown to elucidate much of the pathology associated with T1D and T2D. A universal theme that emerges from β-cell miRNA biology is the specific and careful targeting of gene regulatory networks that control glucose homeostasis and β-cell survival and function. Supplementing the role of applying extensive miRNA profiles to predict the onset of diabetes, uncovering the upstream regulation and downstream targets of pancreatic miRNAs can help foster the development of novel clinical therapies that modulate the expression and activity of these miRNAs and potentially restore a normal glucose homeostasis and β-cell function.

## Figures and Tables

**Table 1:** Circulating microRNA profile changes reported in clinical study on diabetic patients.

Study Design		Source	miRNA Detection Methods	miRNA Expression Alteration		Reference
Non-Diabetic Control	Diabetes Patients					
**Type 1 Diabetes Mellitus**						
54	54	Serum	miRNA microarray, confirmed by qRT-PCR	Up	miR-24, miR-25, miR-26a, miR-27a, miR-27b, miR-29a, miR-30a-5p, miR-148a, miR-152, miR-181a, miR-200a, miR-210,	27
7	10		miRNA qPCR platform	Up	let-7e, let-7g, miR-18a, miR23b, miR-24, miR-25, miR-30e, miR-93, miR-103a, miR-125a, miR-140, miR-144, miR-182, miR-183, miR-192, miR-214, miR-221, miR-222, miR-324–3p, miR324–5p, miR-331, miR-345, miR-377, miR-454, miR-500a, miR-502, miR1468,	28
				Down	miR-100, miR-154, miR-490, miR-630, miR-636, miR-639, miR-675, miR-720	
40	40		qRT-PCR	Up	miR-144, miR-222, miR-345, miR-454	
10	22			Up	miR-181a	31
51	38	Plasma		Down	miR-375	33
79	68			Up	miR-375	32
27	16			Up	miR-21, miR-210	30
				Up	miR-21, miR-24, miR-30d, miR-34a, miR-126, miR146, miR-148a, miR-375	29
**Type 2 Diabetes Mellitus**						
24	24	Serum	qRT-PCR	Up	miR-571, miR-661, miR-770–5p, miR892b, miR-1303	47
82	101			Up	miR-15b, miR-146b, miR-486	46
20	24			Down	let-7i, miR-23a, miR-96, miR-186, miR-191, miR-192, miR-486	44
19	18			Up	miR-9, miR-29a, miR-30d, miR34a, miR-124a, miR-146a, miR-375	42
100	100			Down	miR-126	36
40	56			Down	miR-146a	45
80	80	Plasma	miRNA microarray, confirmed by qRTPCR	Up	miR-28–3p	38
				Down	miR-15a, miR-29b, miR-126, miR-223	
20	20		qRT-PCR	Down	miR-126	37
136	193			Down	miR-126–3p	39
90	90			Up	miR-146a	43
30	30			Down	miR-126	40
107	193			Down	miR-21–5p, miR-126–3p	41
20	61			Down	miR-191, miR-200b	49
27	31			Up	miR-21, miR-24, miR-30d, miR-34a, miR-126, miR146, miR-148a, miR-375	29
46	50	Blood	miRNA microarray, confirmed by qRTPCR	Up	miR-150, −192, −27a, −320a, and −375	68
				Down	miR-17, miR-92a, miR-130a, miR-195, miR-197, miR-509–5p, miR-652	
15	21			Up	miR-29a, miR-144, miR-150, miR-192, miR-320	34
				Down	miR-15a, miR-30d, miR-182	
24	24		qRT-PCR	Down	miR-15a	50
46	127	Platelet	qRT-PCR	Down	miR-103b	51

**Table 2:** Circulating microRNA profile changes reported in animal models of diabetes mellitus.

Animal Models	Source	miRNA Detection Methods	miRNA Expression Alteration		Reference
**T1DM Model**					
Streptozotocin (STZ)-induced mice	Plasma	qRT-PCR	Up	miR-375	52
Non-obese diabetic (NOD) mice					
**T2DM Model**						
High fat diet (HFD), STZ-induced rats	Blood	miRNA microarray, confirmed by qRT-PCR	Up	miR-29a, miR-144, miR-150, miR-192, miR-320a	34
			Down	miR-30d, miR-146a, miR-182	
Zucker diabetic fatty (ZDF) rats	Plasma	qRT-PCR	Up	miR-122, miR-133, miR-210 and miR-375	54
			Down	miR-140, miR-151–3p, miR-185, miR-203, miR-434–3p, miR-450a	
db/db mice	Serum	qRT-PCR	Up	miR-15b, miR-146b, miR-486	46

**Table 3:** Pancreatic islets microRNA profile changes reported in clinical study on diabetic patients.

Study Design		miRNA Detection Methods	miRNA Expression Alteration		References
Non-Diabetic Donors	Diabetes Patients				
**Type 1 Diabetes Mellitus**					
4	2	qRT-PCR	Up	miR-125a-5p	59
**Type 2 Diabetes Mellitus**					
10	5	qRT-PCR	Up	miR-124a	60
9	11	Global profilingTaqMan array, comfirmed by qRT-PCR	Up	miR-187	61
10	9	qRT-PCR	Down	miR-7a	62
19	12		Down	miR-184	63
3	4	Global miRNA Seq	Up	miR-187, miR-216a, miR-589	64
			Down	miR-7–1, miR-7–3, miR-23c, miR-30a, miR-369, miR-487a, miR-487b, miR-495, miR-539, miR-544a, miR-656, miR-4716	
14	10	qRT-PCR	Down	miR-136, miR-369, miR411, miR-432, miR-487a, miR-487b, miR-655, miR-656	

**Table 4:** Pancreatic islets microRNA profile changes reported in animal models of diabetes mellitus.

Animal Models	miRNA Detection Methods	miRNA Expression Alteration		Reference
**T1DM Model**				
Streptozotocin (STZ)induced mice	miRNA microarray, confirmed by qRT-PCR	Down	let-7f-5p, let-7b-5p, let-7a-5p, miR-7b-5p, miR-7a-5p, miR-26a-5p, miR-26b-5p, miR-27a-3p, miR-148b-3p	65
Non-obese diabetic (NOD) mice	qRT-PCR	Down	miR-26a-5p	66
**T2DM Model**				
Goto-Kakizaki (GK) rats	miRNA microarray	Up	let-7i*, miR-7b, miR-124, miR-127, miR-130a, miR-132, miR-136*, miR-142–3p, miR-142–5p, miR-152, miR-199a*−3p, miR-199a-5p, miR-212,miR −335, miR-369–3p, miR-376a, miR-376a*, miR-376b-3p, miR-376c, miR-409–3p, miR-410, miR-411, miR433, miR-434	68
		Down	miR-28*, miR-216, miR-217, miR-493, miR-503, miR-708	
High fat diet (HFD), STZ-induced Rats	miRNA microarray, confirmed by qRT-PCR	Up	miR-29a, miR-144, miR-150, miR-192, miR-320a	34
		Down	miR-30d, miR-146a, miR-182	
	qRT-PCR	Up	miR-141, miR-200a, miR-200b, miR-200c, miR-429	69
	in situ hybridization	Up	let-7b	70
		Down	miR-30d	
	miRNA microarray	Up	miR-10a, miR-10b, miR-21, miR-22*, miR-34a, miR-34b-5p, miR-34c, miR-99a, miR100, miR-126–3p, miR-132, miR-139–5p, miR-143, miR-146a, miR-146b, miR-152, miR-181c, miR-195, miR-199a-3p, miR-199a-5p, miR-199b*, miR-212, miR-320, miR-322, miR-337–5p, miR-365, miR-455*, miR-497, miR-676, miR-721, miR-802, miR-1224	72
		Down	miR-23b, miR-26a, miR-27b, miR-30e, miR-30e*, miR-30d, miR-31, miR-103, miR-129–3p, miR-129–5p, miR-184, miR203, miR-204, miR-210, miR-301a, miR-324–3p,miR-324–5p, miR-325, miR-328, miR-331–3p, miR-338–3p, miR-341, miR-374, miR-378, miR-381, miR-383, miR384–5p, miR-434–3p, miR-652, miR-872	
	qRT-PCR	Up	miR-21, miR-132, miR-199a-3p, miR-199a5p	
		Down	miR-184, miR-203, miR-210, miR-383	
		Up	miR-34a, miR-146	71
		Down	miR-338–3p	73
		Down	miR-7a	62
		Down	miR-184	63
Diet induced obesity (DIO) mice	miRNA microarray	Up	let-7d*, miR-7a-1*, miR-34c, miR-101b, miR-125a-3p, miR-130b*, miR-132, miR152, miR-182, miR-193, miR-200c*, miR-205, miR-211, miR-216b, miR-221, miR322, miR-323–3p, miR-337–3p, miR-362–5p, miR-380–3p, miR-433, miR-455*, miR-484, miR-485*, miR-494, miR-540–3p, miR-615–3p, miR-670, miR-671–5p, miR-680, miR-702, miR-705, miR-714, miR-770–3p, miR-802, miR-1224, miR-1894–5p, miR-1897–5p, miR-1904, miR-1906	72
		Down	let-7b*, miR-10a, miR-24–1*, miR-28, miR29a*, miR-30b*, miR-30c-1*, miR-31*, miR-32, miR-33, miR-100, miR-148a*, miR-181d,miR-184, miR-199a-3p, miR202–3p, miR-203, miR-210, miR-215, miR-218, miR-223, miR-301b, miR-328, miR-335–5p, miR-344b, miR-378, miR-383, miR-384–5p, miR-539–5p, miR-541, miR-543, miR-676, miR-690, miR-697, miR-700, miR-1187, miR-1198–5p, miR-1892	
	qRT-PCR	Up	miR-132, miR-199a-3p, miR-199a-5p	
		Down	miR-184, miR-203, miR-210, miR-383	

**Table 5:** Role of miRNAs in diabetes.

miRNA	Models Used	Function	References
miR-375	MIN6, Nit-1, INS1E	Inhibits GSIS by targeting Mtpn and blocking the fusion and exocytosis of insuiln vesicles at β-cell membrane; also possibly downregulates NF-κB activity. Reduces insulin production and β-cell proliferation by targeting PDK1 and inhibiting PI3K signaling axis	58, 82
miR-124a	MIN6	Increases basal insuline secretion but decreases GSIS by upregulating SNAP25, Rab3A, and synapsin-1A while decreasing Rab27A and Noc2. Rab27A is a direct target.	79
miR-96	MIN6	Inhibits GSIS by upregulating the expression of granuphilin and decreasing Noc2 levels	79
miR-145	MIN6	Decreases GSIS by targeting ABCA1 and decreasing the efflux of cholesterol from the β-cell	81
miR-33	MIN6	Decreases GSIS by targeting ABCA1 and causing accumulation of cholesterol in the β-cell	80
miR-7	MIN6	Negative regulator of GSIS by directly regulating genes involved in distal stages of the fusion of the insulin vesicle with cell membrane and interaction with ternary SNARE complex.	62
miR-204	INS-1, MIN6	Downregulates insulin transcription by directly targeting insulin transcription factor MAFA. Also decreases insulin secretion by targeting GLP1R.	83, 84
miR-21	INS-1, MIN6	Promotes β-cell proliferation. Overexpression increases β-cell proliferation but also activates apoptosis, impairing net β-cell survival. Also downregulates GSIS via indirect inhibition of VAMP2 but does not affect basal insulin secretion	85, 86
miR-34a	INS-1, MIN6	Promotes β-cell death by downregulating SIRT1 and enhancing p53-mediated apoptosis	85, 86
miR-200	MIN5, INS-1E	Contributes to β-cell apoptosis by downregulating antiapoptotic and stress-resistance pathways. Also activates Trp53 pathway and concomitant expression of pro-apoptotic genes.	87

## Abbreviations

ABCA1: ATP-binding Cassette transporter A1
DIO: Diet Induced Obesity
DM: Diabetes Mellitus
GK: Goto-Kakiz
GLP1R: Glucagon-like Peptide 1 Receptor
GSIS: Glucose-stimulated Insulin Secretion
HFD: High Fat Diet
IDF: International Diabetes Federation
miRISC: miRNA-induced Silencing Complex
miRNAs: microRNAs
Mtpn: Myotrophin
NOD: Non-obese Diabetic
PDK1: 3’-phosphoinositide-dependent Protein Kinase-1
PI3K: Phosphatidylinositol 3-kinase
pre-miRNAs: precursor miRNAs
pri-miRNAs: primary miRNAs
RBPs: RNA Binding Proteins
SIRT1: Silent Information Regulator
STAT3: Signal Transducer and Activator of Transcription 3
STZ: Streptozotocin
T1DM: Type 1 Diabetes Mellitus
T2DM: Type 2 Diabetes Mellitus
TXNIP: Thioredoxin-Interacting Protein
ZDF: Zucker Diabetic Fatty
